# Can periodontitis affect the COVID-19 severity, symptoms, hospital stay, and mortality? A case–control study

**DOI:** 10.3389/fpubh.2024.1421380

**Published:** 2024-09-06

**Authors:** Shravya Macherla, Aditi Chopra, Venkitachalam Ramanarayanan, Rajat Subra Das, Rupesh Garg

**Affiliations:** ^1^Department of Periodontology, Manipal College of Dental Sciences, Manipal, Manipal Academy of Higher Education, Manipal, Karnataka, India; ^2^Department of Public Health Dentistry, Amrita School of Dentistry, Amrita Vishwa Vidyapeetham, Kochi, Kerala, India

**Keywords:** coronavirus disease, COVID-19, periodontitis, periodontal disease, SARS-CoV-2

## Abstract

**Background/purpose:**

Poor oral hygiene and periodontal disease have been identified as potential risk factors for the coronavirus disease 2019 (COVID-19). The present study aimed to determine the association between periodontitis and COVID-19 severity, nature of symptoms, mortality, and hospital stay.

**Methods:**

In total, 163 COVID-19-positive patients (men: 93; women: 70) were categorized into two groups: the control group, consisting of 120 patients with asymptomatic or mild symptoms, and the case group, consisting of 43 patients with moderate-to-severe symptoms. The severity of periodontal disease, oral hygiene status (OHI), pocket depth (PD), bleeding on probing (BOP), number of decayed/missing/filled teeth, mortality, duration of stay in the hospital, oxygen requirement, and nature of COVID-19 symptoms were assessed in both groups. The association between periodontitis and COVID-19 was analyzed with other confounding factors such as age, sex, comorbidities, oral hygiene, and smoking status.

**Results:**

The presence of periodontitis increases the severity of COVID-19 by 3.7 times (*p* = 0.002). A statistically significant difference was noted for symptoms such as dizziness (*p* = 0.036), running nose/cold (*p* = 0.009), and headache (*p* = 0.005) in the presence of periodontitis. The risk estimate for death associated with periodontitis was 1.03. Additionally, the average duration of stay was longer for individuals with periodontitis than for those in the control group.

**Conclusion:**

There is a positive association between periodontal disease and COVID-19. Periodontitis increases the severity of COVID-19 and alters the symptoms. Hence, periodontal disease management should be an integral part of managing patients with coronavirus infection.

## Introduction

The coronavirus disease 2019, referred to as COVID-19, is caused by SARS-CoV-2. SARS-CoV-2 is a zoonotic virus, similar to the Middle East respiratory syndrome coronavirus (MERS-CoV) and the SARS-CoV-2 (1, 2). The human-to-human transmission primarily occurred via oral and respiratory droplets, through both direct contact and non-contact modes (2–7). As of 14 May 2023, over 766 million confirmed cases and over 6.9 million deaths have been reported globally. Many variants of SARS-CoV-2 have been discovered across the globe causing myriads of symptoms (8, 9). Some common symptoms of SARS-CoV-2 infection are fever, sore throat, fatigue, body aches, and loss of taste and smell. In severe cases of COVID-19, pneumonia, shortness of breath, need for oxygen or ventilation, and multi-organ failure have been reported (10, 11). Many studies have found that the severity of COVID-19 increases in individuals with comorbidities such as hypertension, diabetes mellitus, cardiovascular disease, respiratory disorders, immunocompromised conditions, and in individuals undergoing radiotherapy and chemotherapy (12–14).

Poor oral hygiene and periodontal disease, an inflammatory condition affecting the soft tissues around the teeth, have recently been associated with many respiratory diseases, including COVID-19 infection (15–25). This association is attributed to the influx of periodontal pathogens and proinflammatory cytokines into the systemic circulation during periodontitis (21–23). Additionally, inflamed periodontal tissues have been identified as a source of increased presence of angiotensin-converting enzyme 2 (ACE2). The increased ACE2 allows the attachment of viral particles, promoting their spread from the oral cavity into the systemic circulation (22). Many studies have established the association between periodontitis and COVID-19 and confirmed that the probability of acquiring COVID-19 is 2.81 times higher in patients with periodontitis (18–25). The risk of COVID-19 also increases as periodontal inflammation increases with an OR of 11.75 for severe periodontitis and an OR of 17.65 for gingivitis (18). Periodontal disease has also been linked to many COVID-19 complications, including death, increased ICU admission, and the need for assisted ventilation (25). Periodontitis has been associated with a 4-fold increase in hospitalization (OR = 4.72); a 6-fold increase in the need for assisted breathing (OR = 6.24); and a more than 7-fold increase in death (OR = 7.51) (20). However, the association between the severity of periodontitis to the nature and severity of COVID-19 has not been explored. With this background, the present study aims to determine the association of periodontitis with the severity of COVID-19. The main objectives of the study are as follows:

1. To correlate and compare the severity of periodontitis with the severity of COVID-19 symptoms, ranging from asymptomatic to mild, and moderate-to-severe symptoms.
2. To assess the relationship between oral hygiene status (plaque and calculus scores), bleeding on probing (BOP), and alveolar bone loss in individuals with mild, moderate, and severe COVID-19 symptoms.
3. To evaluate the effect of age, sex, smoking, and the presence of any comorbidities on the association between COVID-19 and periodontitis.
4. To determine the association between periodontitis, mortality rates, and length of hospital stay for COVID-19 patients.

## Materials and methods

This case–control study was conducted at Manipal College of Dental Sciences, Manipal, Karnataka, India, from December 2019 to January 2022 following the Declaration of Helsinki (as revised in 2013), STROBE, and SAGER guidelines. The study was initiated after approval from the institutional review committee of Kasturba Medical College and Kasturba Medical Hospital (IEC no: IEC2-107/2022; Clinical Trial Registry India registration no: CTRI/2022/08/045088).

### Sample size

The sample size was determined based on a previous study by Mouraf et al. aiming for 80% power (25). Based on the above calculations, a total sample size of 144 was required (case group = 24 and control group =120).

### Screening, eligibility criteria for recruitment, and grouping

The medical and dental records of COVID-19-positive patients were obtained from the Office of Medical Superintendent, Kasturba Medical College, Manipal, from the year 2019 to 2021. A total of 3,392 medical records were screened to note the presence and absence of COVID-19 based on positive reverse transcription–polymerase chain reaction (RT-PCR) reports. Both verbal consent and written informed consent were taken. All the COVID-19-positive patients were further screened based on specific inclusion and exclusion criteria as follows:

#### Inclusion criteria

1. Both male and female participants aged above 18 years with a history of COVID-19 infection and having a previous dental record were included in the study.

#### Exclusion criteria

1. Subjects < 18 years of age.
2. Edentulous patients (with complete loss of teeth).
3. COVID-19-positive patients who were pregnant and lactating at the time of coronavirus infection.

The following data were retrieved from the medical records of each patient who fulfilled the eligibility criteria: the date of the RT-PCR test; the date of onset of COVID-19 infection; and the signs and symptoms of COVID-19. Based on the signs and symptoms, all participants were categorized into asymptomatic, mild, moderate, and severe as per the ICMR guidelines into two groups: case (participants with moderate-to-severe symptoms of COVID-19) and control groups (participants with mild-to-asymptomatic symptoms of COVID-19) (26, 27).

The following data were recorded for both groups: age (in years), sex (male/female), weight (in kilograms) and height (in centimeters), smoking status (yes/no), frequency of brushing, use of interdental aids or mouthwash (yes/no), presence of any comorbidities, hospital stay after COVID-19, and partial pressure of oxygen (PO_2_). The dental records of all the participants were checked by another investigator (SM) who was blinded to the COVID-19 severity and grouping of participants. The dental records were checked for the following data: presence or absence of bleeding on probing (BOP) or percentage of site with BOP; amount of plaque and calculus scores; mean pocket depth (PPD) (in millimeters); and clinical attachment loss (CAL) (in millimeters). The radiographic assessment of bone loss (in millimeters) and the presence/absence of decayed, missing, and filled teeth was noted. The extent of bone loss was measured from the cemento-enamel junction (CEJ) to the apex to the root (in millimeters) using ImageJ software (online version) (28, 29). Based on the average PPD mentioned in the dental records, participants with an average PPD of < 4 mm and no interdental bone loss (BL) were considered as the non-periodontitis group (*N* = 63). Those with an average PPD of more than 4 mm and having two or more teeth with interdental/interproximal bone loss were classified as the case group (periodontitis group; *n* = 100). The periodontitis group was further divided based on the extent of interdental bone loss according to the 2017 periodontal disease classification as Stage I/Stage II/Stage III/Stage IV (28, 29).

### Statistical analysis

All data were entered manually, coded, and proofread for entry errors by two investigators (AC and SM). The data were subjected to statistical analysis using Statistical Package for Social Sciences (SPSS v 20.0, IBM). Descriptive statistics such as frequencies and percentages for categorical variables and the mean and standard deviation (SD) for continuous variables were calculated. To assess the relationship between exposure (severity of periodontitis) and outcome (severity of COVID-19), OR with 95% CI was calculated. Multiple linear regression was performed to predict the effect of the independent variables [age, sex, comorbidities, oral hygiene index (OHI), and smoking] on the association between periodontitis and COVID-19 severity. For all the statistical tests, a *p*-value of < 0.05 was considered to be statistically significant, keeping α error at 5% and β error at 20%.

## Results

**Figure 1:**
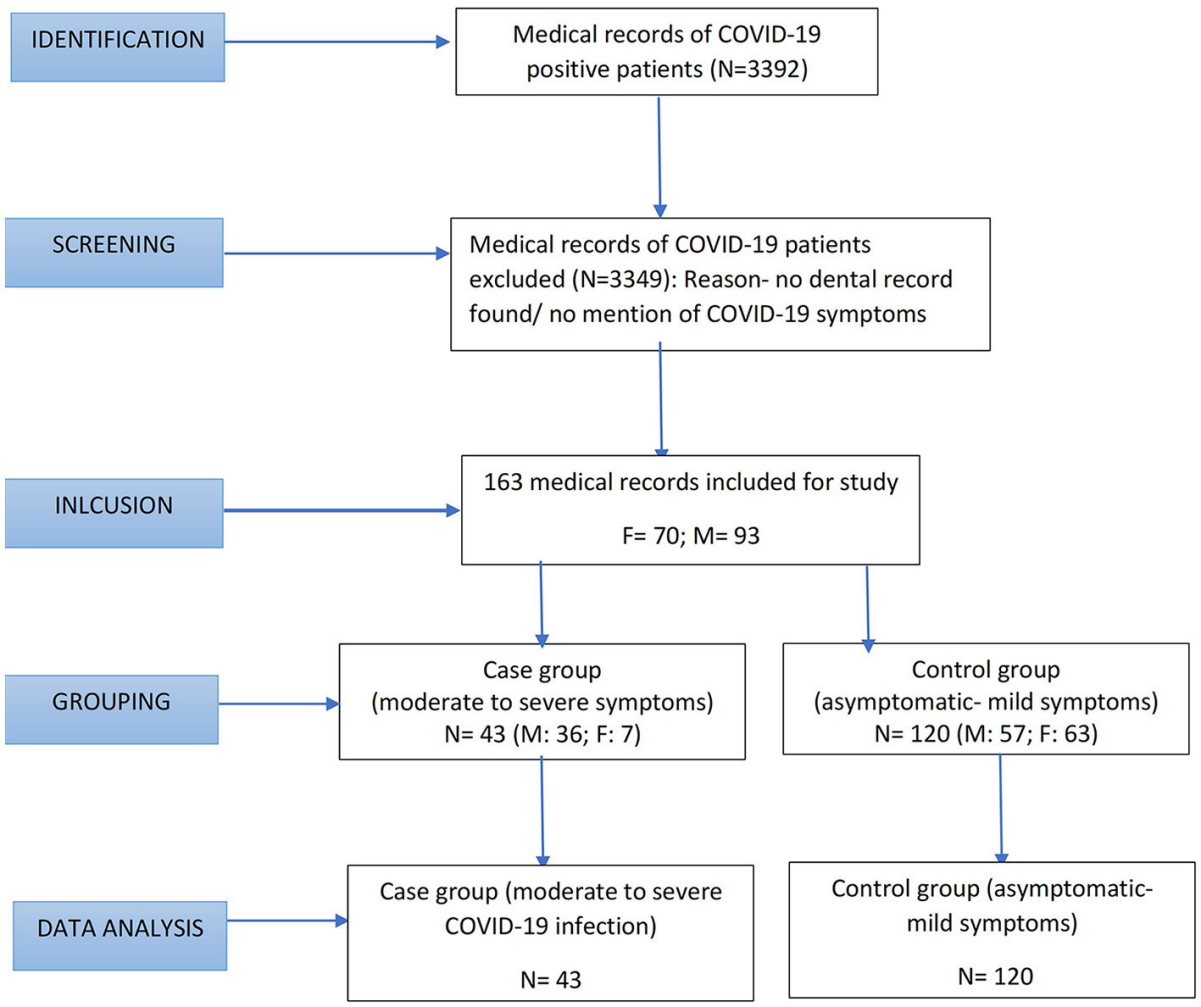
Participant flow diagram.

A total of 3,992 medical records were initially screened for the presence of COVID-19 by checking for a positive RT-PCR report or signed medical diagnosis of COVID-19. Of this, 3,512 records were included and checked for the presence of dental records. Out of 3,512 records, 3,349 were excluded as their dental records were not available. Additionally, 32 records were excluded as the symptoms of COVID-19 or its severity were not mentioned. Finally, medical records of 163 patients were included in the study (Figure 1). Among the 163 participants, 120 participants were in the control group and 43 in the case group. The symptom-wise distribution was as follows: 16 were asymptomatic (13.3%), 104 showed mild symptoms of COVID-19 (86.7%), 21 had moderate symptoms of COVID-19 (48.8%), and 22 had severe (51.2%). The descriptive data and the results of the regression analysis to assess the association between periodontitis and COVID-19 severity based on age, sex, comorbidities, and oral hygiene status are discussed in detail in the following paragraphs (Tables 1–4).

**Table 1:** Demographic characteristics of study participants and their association with COVID-19 and periodontal disease severity.

**Demographic variables**		**COVID-19 severity**			**Periodontal disease severity**		
		**Cases (moderate/severe COVID-19)**	**Controls (asymptomatic/mild COVID-19)**	***p*-value**	**Periodontitis**	**Non-periodontitis**	***p*-value**
Age	Mean ± SD	53.70 ± 19.13	40.67 ± 17.34	**< 0.001** ^*^	Stage I (*n =* 16): 39.31 ± 16.74 Stage II (*n =* 47): 44.02 ± 15.96 Stage III (*n =* 15): 61.27 ± 16.89 Stage IV (*n =* 22): 69.00 ± 11.44	32.60 ± 10.72	**0.044** ^*^
Sex	Males	36 (83.7%)	57 (47.5%)	**< 0.001** ^*^	63 (63.0%)	30 (47.6%)	0.053
	Females	7 (16.3%)	63 (52.5%)		37 (370%)	33 (52.4%)	
Smokeless tobacco	Present	6 (14.0%)	7 (5.8%)	0.092	9 (9.0%)	4 (6.3%)	0.5438
	Absent	37 (86.0%)	113 (94.2%)		91 (91.0%)	59 (93.7%)	
Smoking tobacco	Present	2(4.7%)	10 (8.3%)	0.428	8 (8.0%)	4 (6.3%)	0.694
	Absent	41 (95.3%)	110 (91.7%)		92 (92.0%)	59 (93.7%)	
Medical history—Endocrine (Diabetes Mellitus)	Present	20 (46.5%)	33 (27.7%)	**0.036** ^*^	44 (44.4%)	9 (14.3%)	**< 0.001** ^*^
	Absent	23 (53.5%)	86 (72.3%)		55 (55.6%)	54 (85.7%)	

^*^Significant at a *p-*value of < 0.05. The *p*-value in bold indicates statistically significant results.

### Sex and age

The group-wise distribution showed that 83.7% of the participants in the case group were men and 16.3% were women. The odds of having a higher severity of COVID-19 was 5.68 (*p*-value: 0.001) in men (Table 1, Supplementary Tables S1–S9). Upon comparison of sex with the presence or absence of periodontitis in the case and control groups, a statistically significant difference was seen between the men and women with a *p-*value of 0.053 (Table 1, Supplementary Table S1). Upon comparing the severity of periodontitis, a statistically significant difference was observed in age across different stages of periodontitis (*p* < 0.001).

### Habits (smoking and smokeless tobacco use)

The percentage of smokers and non-smokers in the case group was 4.7% and 95.3%, respectively. In the control group, 8.3% were smokers, and 91.7% were non-smokers (*p* = 0.428) (Table 1). The association between periodontal status and COVID-19 severity, considering the use of smokeless tobacco and smoking, showed a significant association (OR: 5.85; *p*-value < 0.001) (Supplementary Table S1).

### Endocrine diseases

As endocrine disorders (especially diabetes mellitus) are an established risk factor for periodontal disease and COVID-19 severity, the association was assessed. A statistically significant difference was noted between the groups and COVID-19 severity (*p* = 0.036) with 46.5% of moderate–severe COVID-19 patients reporting a history of endocrine disorders compared to 27.7% of asymptomatic–mild cases. The difference in the severity of periodontal diseases was statistically significant (*p* < 0.001), with 44.4% of periodontitis patients and 14.3% of no-periodontitis patients affected by endocrine disease (Table 1).

### Comorbidities

A statistically significant difference was noted between periodontitis and non-periodontitis groups for all comorbidities except for neurological conditions (Supplementary Table S2). The association of comorbidities with the severity of COVID-19 showed an OR of 3.37 for cardiovascular diseases, an OR of 2.26 for endocrine disorders, an OR of 4.04 for respiratory diseases, and an OR of 6.32 for neurological diseases (Tables 2, 4, Supplementary Table S2). Respiratory (*p-*value: 0.009) and neurological conditions (*p*-value: 0.002) were shown to have a significant influence on the association between periodontitis and COVID-19 with an OR of 4.125 and 5.778, respectively (Table 2).

**Table 2:** Association of comorbidities with COVID-19 severity and periodontal status.

**Periodontal status**	**Comorbidities**		**Cases (moderate/severe COVID-19)**	**Controls (asymptomatic/mild COVID-19)**	**OR (95% CI)**	***p*-value**
Periodontitis	H/o cardiovascular disease	Present	22 (62.9%)	27 (42.9%)	2.25 (0.96–5.26)	0.058
		Absent	13 (37.1%)	36 (57.1%)		
	H/o respiratory disease	Present	11 (31.4%)	6 (10.0%)	4.125 (1.36–12.45)	**0.009** ^*^
		Absent	24 (68.6%)	54 (90.0%)		
	H/o endocrine disease	Present	18 (51.4%)	26 (40.6%)	2.256 (0.96–5.26)	0.058
		Absent	17 (48.6%)	38 (59.4%)		
	H/o neurological disease	Present	4 (11.4%)	0 (0%)	-	**0.005** ^*^
		Absent	31 (88.6%)	65 (100%)		
	H/o other systemic diseases	Present	20 (57.1%)	17 (26.2%)	3.765 (1.58–8.97)	**0.002** ^*^
		Absent	15 (42.9%)	48 (73.8%)		
Non-periodontitis	H/o cardiovascular disease	Present	1 (12.5%)	3 (5.5 %)	2.47 (0.22–27.20)	0.445
		Absent	7(87.5%)	52 (94.5%)		
	H/o respiratory disease	Present	0 (0%)	3 (5.5%)	-	0.498
		Absent	8 (100%)	52 (94.5%)		
	H/o endocrine disease	Present	2 (25.0%)	7 (12.7%)	2.47 (0.22–27.20)	0.445
		Absent	6 (75.0%)	48 (87.3%)		
	H/o neurological disease	Present	2 (25.0%)	3 (5.5%)	5.77 (0.79–41.79)	0.056
		Absent	6(75.0%)	52 (94.5%)		
	H/o other systemic diseases	Present	4 (50.0%)	7 (12.7%)	6.85 (1.38–33.85)	**0.009** ^*^
		Absent	4 (50.0%)	48 (87.3%)		

^*^Significant at a *p*-value of < 0.05. The *p*-value in bold indicates statistically significant results.

### Association of periodontitis and periodontal parameters in the case and control groups

In the case group, 100 participants (81.4%) had periodontitis; whereas 65 participants (54.2%) in the control group had periodontitis. In the case group, periodontitis was absent in 18.6% of participants (*p*-value: 0.002) (Table 3). The staging of periodontal disease showed that 9.8% of the individuals were in Stage I, 28.8% were in Stage II, 9.2% were in Stage III, and 13.5 % were in Stage IV. A statistically significant association was observed between periodontitis and COVID-19 severity (*p* = 0.002^*^, OR = 3.702) (Supplementary Tables S3, S6). A significant association was also noted between periodontal parameters, such as BOP, and COVID-19 severity (*p* = 0.017). The BOP in the case and control groups was 95.3% and 100%, respectively (Supplementary Tables S3, S4).

**Table 3:** Association of periodontal status with COVID-19 severity.

**Periodontal status**	**Cases (moderate/severe COVID-19)**	**Controls (asymptomatic/mild COVID-19)**	**OR (95% CI)**	***p*-value**
Periodontitis	35 (81.4%)	65 (54.2%)	3.7 (1.58–8.64)	**0.002** ^*^
Non-periodontitis	8 (18.6%)	55 (45.8%)		

^*^Significant at a *p*-value of < 0.05. The *p*-value in bold indicates statistically significant results.

### Association of oral hygiene practice and decayed/missing/filled teeth scores in the case and control groups

The oral hygiene status of the participants with regard to plaque (*p* = 0.275) and calculus scores (*p* = 0.370) showed no statistically significant difference between the case and the control groups (Supplementary Table S5). When the DMF scores were compared in the case and control groups, the number of decayed and missing teeth was higher in the case group than in the control group. The total DMF score for the case group was 6.72 ± 7.27, and the control group was 4.90 ± 6.40 (*p-*value: 0.124) (Supplementary Table S6).

### Nature of COVID-19 symptoms, mortality, days of hospital stay, and partial pressure of oxygen

Out of 163 individuals, the following COVID-19 symptoms were recorded: presence of fever (*n* = 108), loss of taste (*n* = 8), loss of smell (*n* = 7), dizziness (*n* = 10), presence of GIT disturbances (*n* = 39), breathlessness (*n* = 35), running nose (*n* = 16), cough (*n* = 61), and headache (*n* = 25). No difference was noted in the COVID-19 symptoms between those with periodontitis and non-periodontitis groups. Of those presenting with fever, 62% had periodontitis and 73% did not have periodontitis (OR = 0.60) (Table 3). Only 5% of individuals with periodontitis and 4.8% with non-periodontitis showed loss of taste; 4% of individuals with periodontitis and 4.8% without periodontitis had reported loss of smell. A statistically significant difference was noted only for dizziness (*p* = 0.036), running nose/cold (*p* = 0.009), and headache (*p* = 0.005) (Table 4).

**Table 4:** Association of COVID-19 symptoms with periodontal status for cases (moderate/severe COVID-19) and controls (asymptomatic/mild COVID-19).

**Symptom**	**Group**	**Periodontal status**	**Symptom**		***p*-value**
			**Present**	**Absent**	
Fever	Cases	Periodontitis	24 (80.0%)	11 (84.6%)	0.721
		Non-periodontitis	6 (20.0%)	2 (15.4%)	
	Controls	Periodontitis	38 (48.7%)	27 (64.3%)	0.103
		Non-periodontitis	40 (51.3%)	15 (35.7%)	
Loss of taste	Cases	Periodontitis	1(100%)	34(81.0%)	0.629
		Non-periodontitis	0 (0%)	8(19.0%)	
	Controls	Periodontitis	4(57.1%)	61(54.0%)	0.871
		Non-periodontitis	3(42.9%)	52(46.0%)	
Loss of smell	Cases	Periodontitis	0 (0%)	35(81.4%)	**< 0.001** ^*^
		Non-periodontitis	0 (0%)	8(18.6%)	
	Controls	Periodontitis	4(57.1%)	61(54.0%)	0.871
		Non-periodontitis	3(42.9%)	52(46.0%)	
Dizziness	Cases	Periodontitis	0.00 (0%)	35(83.3%)	0.034
		Non-periodontitis	1(100%)	7(16.7%)	
	Controls	Periodontitis	3(33.3%)	62(55.9%)	0.192
		Non-periodontitis	6(66.7%)	49(44.1%)	
GI disturbances	Cases	Periodontitis	8(80.0%)	27(81.8%)	0.897
		Non-periodontitis	2(20.0%)	6(18.2%)	
	Controls	Periodontitis	16(55.2%)	49(53.8%)	0.901
		Non-periodontitis	13(44.8%)	42(46.2%)	
Breathlessness	Cases	Periodontitis	16(69.6%)	35(83.3%)	**0.033** ^*^
		Non-periodontitis	7(30.4%)	7(16.7%)	
	Controls	Periodontitis	6(50.0%)	62(55.9%)	0.760
		Non-periodontitis	6(50.0%)	49(44.1%)	
Cold or running nose	Cases	Periodontitis	0(0%)	35(81.4%)	**< 0.001** ^*^
		Non-periodontitis	0(0%)	8(18.6%)	
	Controls	Periodontitis	5(31.3%)	60(57.7%)	**0.048** ^*^
		Non-periodontitis	11(68.8%)	44(42.3%)	
Cough	Cases	Periodontitis	15(83.3%)	20(80.0%)	0.782
		Non-periodontitis	3(16.7%)	5(20.0%)	
	Controls	Periodontitis	22(51.2%)	43(55.8%)	0.622
		Non-periodontitis	21(48.8%)	34(44.2%)	
Headache	Cases	Periodontitis	2(66.7%)	33(82.5%)	0.497
		Non-periodontitis	1(33.3%)	7(17.5%)	
	Controls	Periodontitis	7(31.8%)	58(59.2%)	**0.020** ^*^
		Non-periodontitis	15(68.2%)	40(40.8%)	
Mortality	Cases	Periodontitis	9 Deaths (81.8%)	26 Alive (81.3%)	0.967
		Non-periodontitis	2 Deaths (18.2%)	8 Alive (18.6%)	
	Controls	Periodontitis	1 Death (50.0%)	64 Alive (54.2%)	0.905
		Non-periodontitis	1 Death (50.0%)	54 Alive (45.8%)	

^*^Significant at a *p*-value of < 0.05. The *p*-value in bold indicates statistically significant results.

In participants with moderate-to-severe symptoms of COVID-19, 11 individuals died. Out of 11, 9 (81.8%) had periodontitis, and 2 (18.2%) were without periodontitis. In the case group, the risk estimation of death compared to survival for individuals with periodontitis was found to be 1.038. Similarly, among patients with asymptomatic to mild COVID-19, two deaths were reported: one individual with periodontitis and one without periodontitis. The OR was 0.84 in the control group (Table 4, Supplementary Table S7). The mean duration of stay was longer in the periodontitis group than in the non-periodontitis group. The duration of stay also increased as the stage of periodontitis increased. However, the results were not statistically significant (*p*-value: 0.094) (Supplementary Tables S7, S8). The Po2 was lower in individuals with severe COVID-19 symptoms (*p-*value: 0.007); however, no statistically significant difference was noted between the periodontitis and non-periodontitis groups (*p*-value: 0.109) (Supplementary Tables S7, S8).

A multivariate analysis was conducted using three models. In the first model, the severity of COVID-19 was associated with age and periodontal disease status. The model explained 15.1% of the variance in COVID-19 severity. The risk of severe COVID-19 was doubled for patients with periodontitis; however, this finding was not statistically significant. Patients with periodontitis were 3.7 times more likely to exhibit severe COVID-19 (*p* = 0.002). However, there was no statistically significant association between COVID-19 severity and periodontitis in smokers and non-smokers (OR: 0.48). A third model with the most common risk factors (sex, age, periodontal status, smoking, history with cardiac diseases, respiratory diseases, and endocrine diseases) associated with COVID-19 showed a 33.2% variance. Significant variables contributing to the increased severity of COVID-19 were increased age (OR: 1.02), male sex (OR: 5.77), and the presence of respiratory diseases (OR: 3.00) (Supplementary Table S9).

## Discussion

The present study aimed to assess the association of periodontitis with the severity of COVID-19. The study found a positive association between periodontitis and COVID-19 severity (OR = 3.70). We also noted that all patients with COVID-19 had some form of gingival inflammation and compromised oral hygiene. However, no significant association was observed between periodontal parameters, such as BOP and bone loss, between the case and control groups. Patients with moderate-to-severe forms of COVID-19 have shown more decayed and missing teeth compared to those with mild or asymptomatic COVID-19 patients. These results are similar to previous studies by Moradi et al., which found that poor oral hygiene and the presence of periodontitis were associated with increased severity of COVID-19 symptoms, increased admission to the ICU, and increased mortality (30, 31). However, a recent systematic review found that the symptoms of periodontitis, particularly BOP, were not associated with COVID-19 positivity (OR = 1.1) or mortality (OR = 2.71) but were associated with COVID-19 severity (OR = 3.18) (32). Few studies have also observed an increased risk of dizziness and headaches in patients with periodontitis (33–38). We also noted a statistically significant difference in symptoms such as dizziness, running nose, and headache in the periodontitis group compared to the non-periodontitis group. This could be attributed to the direct connection between the oral cavity to the nose, maxillary sinus, and the neurological system. A recent study by Huang et al. in the Taiwanese population also observed an increased risk of dizziness in patients with periodontitis (33). The authors retrieved data from 2008 to 2013 from the National Health Insurance Research of Taiwan. Patients diagnosed with periodontal disease or dizziness after at least one hospital admission or three outpatient visits were included in the study. In total, 445 patients with periodontal disease and 1,780 healthy controls were selected. Dizziness was significantly higher among the patients with periodontal disease relative to the controls. Periodontal disease is not only a risk factor for dizziness but also an independent risk factor for dizziness. Similarly, few studies and systematic reviews have found a positive association between poor oral hygiene, periodontitis, and respiratory such as respiratory pneumonia, COPD, asthma, and headaches, thus justifying the results of the increased prevalence of respiratory and neurological symptoms in COVID-19 patients with periodontitis (34–38).

The correlation between periodontitis and COVID-19 is attributed to increased systemic oxidative stress and inflammatory burden with increasing severity of periodontitis. Additionally, the presence of ACE2 receptors in the inflamed periodontal tissues also contributes to the presence of coronavirus in the oral cavity and its invasion into the systemic circulation (39, 40). The local inflammatory burden and oxidative stress in the inflamed periodontal tissues must be prevented and managed effectively. This is crucial as the proinflammatory cytokines enter the systemic circulation and increase the inflammatory burden in the body, including the respiratory system (41).

Although our study did not evaluate the effect of oral hygiene maintenance in systemically compromised patients, it is crucial for reducing the severity of coronavirus infections. Therefore, the maintenance of good oral hygiene and the control of periodontal disease is crucial for the effective management of COVID-19. Simple measures such as practicing good oral hygiene and scheduling regular dental visits could help prevent or decrease the severity of COVID-19. Although our study found that increasing severity of periodontitis is associated with more severe COVID-19 symptoms, we would like to highlight some limitations of the study. One key limitation is that the data were collected from patients aged 18 years and older; therefore, the role of periodontitis in young individuals or the pediatric population cannot be commented upon in this study. Our study analyzed patients with asymptomatic and mild COVID-19 symptoms as well as those with moderate-to-severe forms of the disease. Hence, future research studies should compare symptomatic and asymptomatic individuals and their correlation to periodontal disease. Additionally, studies should investigate how COVID-19 affects periodontal outcomes and evaluate the effectiveness of periodontal therapy in COVID-19 patients.

## Conclusion

A positive association exists between periodontitis and the severity of COVID-19. Respiratory and neurological disorders significantly influence the association between periodontitis and COVID-19 severity. Increased severity of periodontitis is associated with higher hospital stays and mortality. Individuals with periodontitis have a higher risk of experiencing dizziness, headaches, and running noses than those without periodontitis. An interdisciplinary approach involving the management of periodontal disease and maintaining good oral hygiene for the effective management of COVID-19 should be further explored.

## Data Availability

The original contributions presented in the study are included in the article/Supplementary material, further inquiries can be directed to the corresponding author.

## References

[1] Chan JF Yuan S Kok KH To KK Chu H Yang J. A familial cluster of pneumonia associated with the 2019 novel coronavirus indicating person-to-person transmission: a study of a family cluster. Lancet. (2020) 395:514–23. 10.1016/S0140-6736(20)30154-9 31986261 PMC7159286

[2] Liu J Liao X Qian S Yuan J Wang F Liu Y. Community transmission of severe acute respiratory syndrome coronavirus 2, Shenzhen, China, 2020. Emerg Infect Dis. (2020) 26:1320–3. 10.3201/eid2606.200239 32125269 PMC7258448

[3] Feng L Li J Yao MJ Sun NL Xu JN Su C et al. Interpretation of policies for group standards and the practice of group standardizations in the Chinese Preventive Medicine Association. Zhonghua Liu Xing Bing Xue Za Zhi. (2019) 40:371–375. 10.3760/cma.j.issn.0254-6450.2019.04.001 31006193

[4] Malik YS Sircar S Bhat S Sharun K Dhama K Dadar M. Emerging novel coronavirus (2019-nCoV)-current scenario, evolutionary perspective based on genome analysis and recent developments. Vet Q. (2020) 40:68–76. 10.1080/01652176.2020.1727993 32036774 PMC7054940

[5] Andersen KG Rambaut A Lipkin WI Holmes EC Garry RF. The proximal origin of SARS-CoV-2. Nat Med. (2020) 26:450–2. 10.1038/s41591-020-0820-9 32284615 PMC7095063

[6] Gralton J Tovey ER McLaws ML Rawlinson WD. Respiratory virus RNA is detectable in airborne and droplet particles. J Med Virol. (2013) 85:2151–9. 10.1002/jmv.23698 23959825

[7] Zhu N Zhang D Wang W Li X Yang B Song J. China novel coronavirus investigating and research team. A novel coronavirus from patients with pneumonia in China, 2019. N Engl J Med. (2020) 382:727–733. 10.1056/NEJMoa2001017 31978945 PMC7092803

[8] World Health Organization (WHO). Emergency Situational Updates: Weekly epidemiological update on COVID-19. (2023). Available at: https://www.who.int/publications/m/item/weekly-epidemiological-update-on-covid-19-−18-may-2023 (accessed September 4, 2023).

[9] Singh H Dahiya N Yadav M Sehrawat N. Emergence of SARS-CoV-2 new variants and their clinical significance. Can J Infect Dis Med Microbiol. (2022) 2022:7336309. 10.1155/2022/7336309 35669528 PMC9167142

[10] Pfützner A Lazzara M Jantz J. Why do people with diabetes have a high risk for severe COVID-19 disease? A dental hypothesis and possible prevention strategy. J Diabetes Sci Technol. (2020) 14:769–71. 10.1177/1932296820930287 32506937 PMC7673189

[11] Thakur B Dubey P Benitez J Torres JP Reddy S Shokar N. systematic review and meta-analysis of geographic differences in comorbidities and associated severity and mortality among individuals with COVID-19. Sci Rep. (2021) 11:8562. 10.1038/s41598-021-88130-w 33879826 PMC8058064

[12] Rodilla E Saura A Jiménez I Mendizábal A Pineda-Cantero A Lorenzo-Hernández E. Association of hypertension with all-cause mortality among hospitalized patients with COVID-19. J Clin Med. (2020) 9:3136. 10.3390/jcm9103136 32998337 PMC7650567

[13] Li G Chen Z Lv Z Li H Chang D Lu J. Diabetes mellitus and COVID-19: associations and possible mechanisms. Int J Endocrinol. (2021) 2021:7394378. 10.1155/2021/7394378 33859687 PMC8025139

[14] Sivaraman K Chopra A Narayana A Radhakrishnan RA. A five-step risk management process for geriatric dental practice during SARS-CoV-2 pandemic. Gerodontology. (2021) 38:17–26. 10.1111/ger.12499 32978832 PMC7537327

[15] Herrera D Serrano J Roldán S Sanz M. Is the oral cavity relevant in SARS-CoV-2 pandemic? Clin Oral Investig. (2020) 24:2925–30. 10.1007/s00784-020-03413-2 32577830 PMC7309196

[16] Botros N Iyer P Ojcius DM. Is there an association between oral health and severity of COVID-19 complications? Biomed J. (2020) 43:325–7. 10.1016/j.bj.2020.05.016 32713780 PMC7258848

[17] Takahashi Y Watanabe N Kamio N Kobayashi R Iinuma T Imai K. Aspiration of periodontopathic bacteria due to poor oral hygiene potentially contributes to the aggravation of COVID-19. J Oral Sci. (2020) 63:1–3. 10.2334/josnusd.20-0388 33177276

[18] Anand PS Jadhav P Kamath KP Kumar SR Vijayalaxmi S Anil S. case-control study on the association between periodontitis and coronavirus disease (COVID-19). J Periodontol. (2022) 93:584–90. 10.1002/JPER.21-0272 34347879

[19] Mishra S Gupta V Rahman W Gazala MP Anil S. Association between periodontitis and COVID-19 based on severity scores of HRCT chest scans. Dent J (Basel). (2022) 10:106. 10.3390/dj10060106 35735648 PMC9222103

[20] Baima G Marruganti C Sanz M Aimetti M Romandini M. Periodontitis and COVID-19: biological mechanisms and meta-analyses of epidemiological evidence. J Dent Res. (2022) 101:1430–40. 10.1177/00220345221104725 35774019

[21] Shen Z Xiao Y Kang L Ma W Shi L Zhang L. Genomic diversity of severe acute respiratory syndrome-coronavirus 2 in patients with coronavirus disease 2019. Clin Infect Dis. (2020) 71:713–720. 10.1093/cid/ciaa203 32129843 PMC7108196

[22] Yamasaki K Kawanami T Yatera K Fukuda K Noguchi S Nagata S. Significance of anaerobes and oral bacteria in community-acquired pneumonia. PLoS ONE. (2013) 8:e63103. 10.1371/journal.pone.0063103 23671659 PMC3646017

[23] Del Valle DM Kim-Schulze S Huang HH Beckmann ND Nirenberg S Wang B. An inflammatory cytokine signature predicts COVID-19 severity and survival. Nat Med. (2020) 26:1636–43. 10.1038/s41591-020-1051-9 32839624 PMC7869028

[24] Cuschieri S. The STROBE guidelines. Saudi J Anaesth. (2019) 13:S31–4. 10.4103/sja.SJA_543_18 30930717 PMC6398292

[25] Marouf N Cai W Said KN Daas H Diab H Chinta VR. Association between periodontitis and severity of COVID-19 infection: a case-control study. J Clin Periodontol. (2021) 48:483–91. 10.1111/jcpe.13435 33527378 PMC8014679

[26] Delang L Neyts J. Medical treatment options for COVID-19. Eur Heart J Acute Cardiovasc Care. (2020) 9:209–14. 10.1177/2048872620922790 32363880 PMC7235633

[27] Agrawal AA. A randomized clinical study to assess the reliability and reproducibility of “Sign Grading System”. Indian J Dent Res. (2011) 22:285–90. 10.4103/0970-9290.84305 21891901

[28] Tonetti MS Greenwell H Kornman KS. Staging and grading of periodontitis: Framework and proposal of a new classification and case definition. J Periodontol. (2018) 89:S159–S172. 10.1002/JPER.18-0006 29926952

[29] Chapple ILC Mealey BL Van Dyke TE Bartold PM Dommisch H Eickholz P. Periodontal health and gingival diseases and conditions on an intact and a reduced periodontium: consensus report of workgroup 1 of the 2017 World Workshop on the classification of periodontal and peri-implant diseases and conditions. J Periodontol. (2018) 89 Suppl 1:S74–84. 10.1002/JPER.17-0719 29926944

[30] Moradi Haghgoo J Torkzaban P Farhadian M Moosavi Sedeh SA. Association between the severity of periodontitis, COVID-19, C-reactive protein and interleukin-6 levels in hospitalized patients: a case-control study. BMC Oral Health. (2023) 23:556. 10.1186/s12903-023-03270-x 37568161 PMC10422752

[31] Costa CA Vilela ACS Oliveira SA Gomes TD Andrade AAC Leles CR. Poor oral health status and adverse COVID-19 outcomes: a preliminary study in hospitalized patients. J Periodontol. (2022) 93:1889–901. 10.1002/JPER.21-0624 35294780 PMC9088593

[32] Qi X Northridge ME Hu M Wu B. Oral health conditions and COVID-19: a systematic review and meta-analysis of the current evidence. Aging Health Res. (2022) 2:100064. 10.1016/j.ahr.2022.100064 35281130 PMC8896863

[33] Huang FM Luo CW Lee SS Ho YC Li YC Chang YC. Relationship between periodontal disease and dizziness in Taiwanese adults: a nationwide population-based cohort study. Medicine. (2023) 102:e32961. 10.1097/MD.0000000000032961 36827024 PMC11309663

[34] Mohammed MMA Almayeef D Abbas D Ali M Haissam M Mabrook R. The association between periodontal disease and chronic migraine: a systematic review. Int Dent J. (2023) 73:481–8. 10.1016/j.identj.2023.04.007 37225630 PMC10350603

[35] Dholakia SB Rao P Talluri S Khan J. The association between migraines and periodontal disease: a systematic review of clinical studies. J Oral Biosci. (2023) 65:137–45. 10.1016/j.job.2023.04.001 37062448

[36] Imsand M Janssens JP Auckenthaler R Mojon P Budtz-Jørgensen E. Bronchopneumonia and oral health in hospitalized older patients. A pilot study. Gerodontology. (2002) 19:66–72. 10.1111/j.1741-2358.2002.00066.x 12542215

[37] Brock M Bahammam S Sima C. The relationships among periodontitis, pneumonia and COVID-19. Front Oral Health. (2022) 2:801815. 10.3389/froh.2021.801815 35128525 PMC8813972

[38] Gomes-Filho IS Cruz SSD Trindade SC Passos-Soares JS Carvalho-Filho PC Figueiredo et al. Periodontitis and respiratory diseases: a systematic review with meta-analysis. Oral Dis. (2020) 26:439–46. 10.1111/odi.13228 31715080

[39] Carmona Loayza DA Lafebre MF. Periodontal disease and COVID-19: Prognosis and potential pathways of association in their pathogenesis. Can J Dent Hyg. (2023) 57:44–51. 36968799 PMC10032643

[40] Zhong M Lin B Pathak JL Gao H Young AJ Wang X. ACE2 and furin expressions in oral epithelial cells possibly facilitate COVID-19 infection via respiratory and fecal-oral routes. Front Med. (2020) 7:580796. 10.3389/fmed.2020.580796 33363183 PMC7758442

[41] Gaeckle NT Pragman AA Pendleton KM Baldomero AK Criner GJ. The oral-lung axis: the impact of oral health on lung health. Respir Care. (2020) 65:1211–20. 10.4187/respcare.07332 32156792

