# Supplementary material for: Can periodontitis affect the COVID-19 severity, symptoms, hospital stay, and mortality? A case–control study
Source: Front Public Health. 2024 Sep 6;12:1421380. doi: 10.3389/fpubh.2024.1421380 (PMC11412828; doi:10.3389/fpubh.2024.1421380)
Supplement: Supplementary file 1 [file Table_1.doc]

**Supplementary Table 1:** **Association of periodontal status with COVID-19 severity based on gender, smokeless tobacco use, and smoking status**

| **Variables** | **Periodontal status** | **Cases (Moderate/Severe COVID-19)** | **Controls (Asymptomatic/Mild COVID-19)** | **OR (95% CI)** | **p-value** |
| --- | --- | --- | --- | --- | --- |
| Males | Periodontitis | 6 (16.7%) | 24 (42.1%) | 3.636 (1.308-10.106 | 0.001* |
|  | Non-periodontitis | 36 (83.3%) | 57 (57.9%) |  |  |
| Females | Periodontitis | 5 (71.4%) | 32 (50.8%) | 2.422 (0.437-13.425 | 0.299 |
|  | Non-periodontitis | 2 (28.6%) | 31 (49.2%) |  |  |
| SLT use Present | Periodontitis | 3 (50%) | 6 (85.7%) | 0.167 (0.01-2.368) | 0.164 |
|  | Non-periodontitis | 3 (50%) | 1 (14.3%) |  |  |
| SLT use Absent | Periodontitis | 32 (86.5%) | 59 (52.2%) | 5.85* (2.12-16.18) | < 0.001* |
|  | Non-periodontitis | 5 (13.5%) | 54 (47.8%) |  |  |
| Smoking present | Periodontitis | 0 (.0%) | 8 (80%) | 2.000 (0.751-5.329) | 0.028* |
|  | Non-periodontitis | 2 (100.0%) | 2 (20.0%) |  |  |
| Smoking absent | Periodontitis | 35 (85.4%) | 57 (51.8%) | 5.424  (2.112-13.932) | <0.001* |
|  | Non-periodontitis | 6 (14.6%) | 53 (48.2%) |  |  |

*** significant at p-value less than 0.05**

**Supplementary Table 2: Distribution of participants with systemic conditions according to the presence and absence of periodontitis**

| **Systemic condition** | | **Periodontal status** | | **Total** | **P- value** |
| --- | --- | --- | --- | --- | --- |
|  |  | **Periodontitis** | **Non-periodontitis** |  |  |
| **MH cardi**  **ovascular** | Present | 49 | 4 | 53 | < 0.001*  OR = 14.75  (4.97 – 43.75) |
|  |  | 50.0% | 6.3% | 32.9% |  |
|  | Absent | 49 | 59 | 108 |  |
|  |  | 50.0% | 93.7% | 67.1% |  |
| **MH endocrine** | Present | 44 | 9 | 53 | < 0.001*  OR = 4.8  (2.13 – 10.78) |
|  |  | 44.4% | 14.3% | 32.7% |  |
|  | Absent | 55 | 54 | 109 |  |
|  |  | 55.6% | 85.7% | 67.3% |  |
| **MH respiratory** | Present | 17 | 3 | 20 | 0.015*  OR = 4.35 (1.22 – 15.56) |
|  |  | 17.9% | 4.8% | 12.7% |  |
|  | Absent | 78 | 60 | 138 |  |
|  |  | 82.1% | 95.2% | 87.3% |  |
| **MH**  **neurological** | Present | 4 | 5 | 9 | 0.284  OR = 0.48  (0.12 – 1.87) |
|  |  | 4.0% | 7.9% | 5.5% |  |
|  | Absent | 96 | 58 | 154 |  |
|  |  | 96.0% | 92.1% | 94.5% |  |
| **MH other systemic conditions** | Present | 37 | 11 | 48 | 0.008*  OR = 2.77 (1.29 – 5.97) |
|  |  | 37.0% | 17.5% | 29.4% |  |
|  | Absent | 63 | 52 | 115 |  |
|  |  | 63.0% | 82.5% | 70.6% |  |
| Total | | 100 | 63 | 163 |  |
|  |  | 100.0% | 100.0% | 100.0% |  |

*** significant at p-value less than 0.05**

**Supplementary Table 3: Association of periodontitis findings (bleeding on probing; calculus and dental plaque factors with severity of COVID-19 (+: plaque or calculus covering less than 1/3 of the tooth surface; ++: plaque or calculus covering more 1/3 but less than 2/3 of the tooth surface; +++: plaque and calculus covering more than 2/3 of the tooth surface.**

| **Periodontal parameters** | | **Group** | | **Total** | **P-value** |
| --- | --- | --- | --- | --- | --- |
|  |  | **Cases (Moderate / Severe COVID-19)** | **Controls (Asymptomatic / Mild COVID-19)** |  |  |
| **Bleeding on probing** | **Present** | 41 | 120 | 161 | **0.017***  **OR = 0.25**  **(0.19-0.33)** |
|  |  | 95.3% | 100.0% | 98.8% |  |
|  | **Absent** | 2 | 0 | 2 |  |
|  |  | 4.7% | .0% | 1.2% |  |
| **Dental plaque** | **+** | 41 | 106 | 147 | **0.275** |
|  |  | 95.3% | 88.3% | 90.2% |  |
|  | **++** | 1 | 12 | 13 |  |
|  |  | 2.3% | 10.0% | 8.0% |  |
|  | **+++** | 1 | 2 | 3 |  |
|  |  | 2.3% | 1.7% | 1.8% |  |
| **Calculus** | **+** | 36 | 89 | 125 | **0.370** |
|  |  | 83.7% | 74.2% | 76.7% |  |
|  | **++** | 6 | 29 | 35 |  |
|  |  | 14.0% | 24.2% | 21.5% |  |
|  | **+++** | 1 | 2 | 3 |  |
|  |  | 2.3% | 1.7% | 1.8% |  |
| **Total** | | 43 | 120 | 163 |  |
|  |  | 100.0% | 100.0% | 100.0% |  |

*** significant at p-value less than 0.05**

**Supplementary Table 4: Mean bone loss (in mm) in the case and control group based on the severity of periodontal disease**

| **Stage of Periodontitis** | **Case group (Mean ± SD)** | **Control group**  **(Mean ± SD)** | **P-value** | **T**  **Df** | **95% CI**  **(Lower- upper limit)** |
| --- | --- | --- | --- | --- | --- |
| Stage 1-II (N=63) | 1.263 ± 1.554 | 2.334 ± 1.117 | 0.0013 | t = 3.4571 df = 42 | 1.695-0.445 |
| Stage III-IV  (N=37) | 3.1282 ± 2.672 | 4.328 ± 2.240 | 0.0495 | t = 2.0842 df = 21 | -2.396 -0.002 |

*** significant at p-value less than 0.05**

**Supplementary Table 5: Association of oral hygiene practices with severity of COVID-19;** *** statistically Significant**

| Oral hygiene practice | | **Group** | | **Total** | **p-value** |
| --- | --- | --- | --- | --- | --- |
|  |  | **Cases (Moderate / Severe COVID-19)** | **Controls (Asymptomatic / Mild COVID-19)** |  |  |
| **OHI brushing** | **Once** | 37 | 85 | 122 | 0.135 |
|  |  | 86.0% | 70.8% | 74.8% |  |
|  | **Twice** | 6 | 34 | 40 |  |
|  |  | 14.0% | 28.3% | 24.5% |  |
|  | **Thrice** | 0 | 1 | 1 |  |
|  |  | 0% | 0.8% | .6% |  |
| **OHI interdental brush use** | **No** | 43 | 120 | 163 | 0.000 |
|  |  | 100.0% | 100.0% | 100.0% |  |
| **OHI mouthwash** | **Yes** | 0 | 2 | 2 | 0.394 |
|  |  | 0% | 1.7% | 1.2% |  |
|  | **No** | 43 | 118 | 161 |  |
|  |  | 100.0% | 98.3% | 98.8% |  |

*** significant at p-value less than 0.05**

**Supplementary Table 6: Number of decayed Missing and filled treated teeth (DMFT) in case and control groups**

| **Variable** | **Cases (Moderate / Severe COVID-19)** | **Controls (Asymptomatic / Mild COVID-19)** | **P- value** |
| --- | --- | --- | --- |
| **DMFT total score** | 6.72 ± 7.27 | 4.90 ± 6.40 | 0.124 |

*** significant at p-value less than 0.05**

**Supplementary Table 7: Comparison of duration of stay in hospital and oxygen saturation in case and control group. * Statistically Significant**

| **Variable** | **Group/severity of COVID-19** | **N** | **Mean** | **Std. Deviation** | **p-value** |
| --- | --- | --- | --- | --- | --- |
| **Duration of hospital stay (in days)** | **Asymptomatic** | **16** | **6.69** | **7.01** | **0.029** |
|  | **Mild** | **104** | **6.25** | **3.09** |  |
|  | **Moderate** | **21** | **9.24** | **5.11** |  |
|  | **Severe** | **22** | **7.14** | **4.78** |  |
|  | **Total** | **163** | **6.80** | **4.22** |  |
|  | **Cases (Moderate/Severe COVID-19)** | **43** | **8.16** | **5.004** | **0.031*** |
|  | **Controls (Asymptomatic/Mild COVID-19)** | **120** | **6.31** | **3.808** |  |
| **po2** | **Asymptomatic** | **4** | **99.00** | **.81** | **< 0.001** |
|  | **Mild** | **67** | **97.13** | **3.18** |  |
|  | **Moderate** | **11** | **94.73** | **3.82** |  |
|  | **Severe** | **10** | **87.50** | **11.49** |  |
|  | **Total** | **92** | **95.88** | **5.61** |  |
|  | **Cases (Moderate/Severe COVID-19)** | **21** | **91.29** | **8.967** | **0.007*** |
|  | **Controls (Asymptomatic/Mild COVID-19)** | **71** | **97.24** | **3.128** |  |

*** significant at p-value less than 0.05**

**Supplementary Table 8: Comparison of continuous variables with different stages of periodontitis. * Statistically Significant**

| **Variable** | **Stage of Periodontitis** | **N** | **Mean** | **Std. Deviation** | **p-value** |
| --- | --- | --- | --- | --- | --- |
| Duration of hospital stay (in days) | Non-periodontitis | 63 | 6.35 | 3.173 | 0.094 |
|  | Stage I | 16 | 6.56 | 3.723 |  |
|  | Stage II | 47 | 7.79 | 5.213 |  |
|  | Stage III | 15 | 8.13 | 4.969 |  |
|  | Stage IV | 22 | 5.23 | 3.891 |  |
|  | Total | 163 | 6.80 | 4.221 |  |
| po2 | Non-periodontitis | 35 | 97.26 | 2.904 | 0.109 |
|  | Stage I | 9 | 96.89 | 1.691 |  |
|  | Stage II | 24 | 93.88 | 8.739 |  |
|  | Stage III | 9 | 97.56 | 1.333 |  |
|  | Stage IV | 15 | 94.27 | 6.273 |  |
|  | Total | 92 | 95.88 | 5.614 |  |

*** significant at p-value less than 0.05**

**Supplementary Table 9: Comparison of continuous variables with different stages of periodontitis.**

| **Variable** | **Stage of Periodontitis** | **N** | **Mean** | **Std. Deviation** | **p-value** |
| --- | --- | --- | --- | --- | --- |
| Duration of hospital stay (in days) | Non-periodontitis | 63 | 6.35 | 3.173 | 0.094 |
|  | Stage I | 16 | 6.56 | 3.723 |  |
|  | Stage II | 47 | 7.79 | 5.213 |  |
|  | Stage III | 15 | 8.13 | 4.969 |  |
|  | Stage IV | 22 | 5.23 | 3.891 |  |
|  | Total | 163 | 6.80 | 4.221 |  |
| po2 | Non-periodontitis | 35 | 97.26 | 2.904 | 0.109 |
|  | Stage I | 9 | 96.89 | 1.691 |  |
|  | Stage II | 24 | 93.88 | 8.739 |  |
|  | Stage III | 9 | 97.56 | 1.333 |  |
|  | Stage IV | 15 | 94.27 | 6.273 |  |
|  | Total | 92 | 95.88 | 5.614 |  |

*** significant at p-value less than 0.05**
